# *Monascus purpureus*-fermented Thai glutinous rice reduces blood and hepatic cholesterol and hepatic steatosis concentrations in diet-induced hypercholesterolemic rats

**DOI:** 10.1186/s12906-015-0624-5

**Published:** 2015-03-28

**Authors:** Anurak Bunnoy, Kanokporn Saenphet, Saisamorn Lumyong, Supap Saenphet, Siriwadee Chomdej

**Affiliations:** Department of Biology, Faculty of Science, Chiang Mai University, Chiang Mai, 50200 Thailand

**Keywords:** Thai red yeast rice, *Monascus purpureus*, Hypocholesterolemic effects, Serum cholesterol, Hepatic lipid, Hepatic mRNA expression, LDL receptor, HMG-CoA reductase, CYP7A1

## Abstract

**Background:**

Red yeast rice (RYR) is a fermented product used as a food supplement to promote blood circulation and lower blood cholesterol levels in eastern Asia. Interestingly, monacolin K is the most active compound in RYR that proved to inhibit HMG-CoA reductase in the cholesterol biosynthesis pathway.

**Methods:**

The hypocholesterolemic effects of oral administration of Thai RYR, produced by fermentation of Thai glutinous rice (*Oryza sativa* L. var. Niaw San-pah-tawng) with *Monascus purpureus* CMU 002U, were determined in normal and hypercholesterolemic rats. The rats were divided into six groups, and fed two different kinds of diet. Groups I-II, normal rats fed with a normal diet (SP-diet), were treated with distilled water (SP-control) and 2.0 g/kg/day of RYR extract (SP-2 g). In Groups III-VI, the rats were rendered hypercholesterolemic by feeding them a high fat and cholesterol diet (HFC-diet), and were treated with distilled water (HFC-control), 1.0 g/kg/day (HFC-1 g), 2.0 g/kg/day (HFC-2 g) of RYR extract, and 5.0 mg/kg/day of rosuvastatin (HFC-rosuvastatin) for 30 days, respectively.

**Results:**

The RYR extract significantly decreased the concentrations of serum total cholesterol and low density lipoprotein cholesterol (LDL-C), atherosclerotic index, LDL-C/HDL-C ratio and hepatic cholesterol levels in both HFC-1 g and HFC-2 g groups (*p <* 0.05) as compared with the HFC-control group, and with no significant change in high density lipoprotein cholesterol (HDL-C) concentrations among all six groups. The reduction of serum TC and LDL-C also paralleled the observed changes in mRNA expressions of the genes involved in cholesterol biosynthesis and homeostasis in the liver. The hypercholesterolemic rats treated with RYR extract were significantly higher in LDLR and HMGR expression, but lower in CYP7A1 expression when compared to the untreated hypercholesterolemic rats (HFC-control) (*p <* 0.05). The hepatic injuries in hypercholesterolemic rats were also obviously alleviated by RYR extract.

**Conclusions:**

The extract of Thai RYR possessed potent hypocholesterolemic and anti-atherogenic activities in diet-induced hypercholesterolemic rats. The possible mechanism involving cholesterol-lowering potential of the extract might contribute to its ability to increase LDL-C endocytosis in hepatocyte and to competitively inhibit HMG-CoA reductase, a key enzyme for cholesterol biosynthesis in liver.

## Background

Hypercholesterolemia is one of metabolic syndromes characterized by the high level of plasma cholesterol. Incidence of hypercholesterolemia is a major public health problem and it continues to increase at an alarming rate. It can significantly increase the risk of developing cardiovascular diseases (CVDs), including atherosclerosis which is the most common cause of morbidity and mortality worldwide [1], accounting for almost 17 million deaths annually, and is still most likely to be the main cause of death in the future [2]. Hypercholesterolemia could be classified as either familial hypercholesterolemia or acquired hypercholesterolemia. Familial hypercholesterolemia is caused by specific genetic abnormalities, especially the mutation of genes encoding LDL receptor in both hepatic and extrahepatic tissues. As a consequence, the level of plasma LDL-C is extremely and constantly high leading to the early development of heart disease [3]. In addition to CVDs, familial hypercholesterolemia also leads to many other complications such as xanthomas, xanthelasmas, and obesity [4]. In contrast to familial hypercholesterolemia, acquired hypercholesterolemia or diet-induced hypercholesterolemia is not inherited. It is characterized by the increased levels of LDL-C as well as decreased levels of HDL-C. This latter type of hypercholesterolemia is more common than the former [5].

The dramatically increase incidence of non-inherited hypercholesterolemia could be attributed to environmental factors such as obesity and dietary factors. Foods that contain high saturated fat and cholesterol are believed to be the most important factor of hypercholesterolemias [6]. In addition, clinical studies have shown that a high cholesterol diet and high levels of lipoproteins, particularly LDL-C, in serum, can notably disturb the cholesterol metabolism in the liver, and may also lead to the development of hepatic steatosis formation or non-alcoholic fatty liver disease (NAFLD) [7]. However, the relationship between cholesterol and low density lipoproteins has been a concern for many years, due to its potential to cause CVDs. Therefore, this issue needs to be resolved urgently. The prevention of CVDs can be addressed in several ways, for example by increasing HDL-C or decreasing total cholesterol and LDL-C intake, associated with eating healthy food [8,9]. Additionally, the use of cholesterol lowering drugs, such as statins, is the most common and widely used method in hypercholesterolemia treatment [10]. However, the unaffordable price and side effects of these drugs make the discovery of more effective and safer alternative products to lower serum cholesterol much more attractive.

Red yeast rice (RYR), a fermented product of yeast (*Monascus purpureus*) in rice, has been recognized as early as 800 A.D., during the Tang Dynasty in China [11]. It has a long history as being used as a preservative and a natural dye for foods. It has also been used as medicine for treating digestive disorder and promoting blood circulation in some Asian countries [12]. Monacolin K, (also known under the names mevinolin or lovastatin) is the major compound occurring in the secondary metabolites of RYR metabolism. It acts as 3-hydroxy-3-methyl-glutaryl-CoA reductase (HMG-CoA reductase) inhibitor [13]. Its ability to inhibit cholesterol biosynthesis in the hepatic cells is due to its competitive inhibitory effect upon HMG-CoA reductase activity [14,15]. In addition to monakolin K, RYR also contains many substances, for instances, fatty acid, palmitic acid, linoleic acid, oleic acid and stearic acid. Those substances have been postulated as the key regulators for serum cholesterol homeostasis [11,16]. Many studies have succeeded in lowering serum cholesterol levels in various species, including: chickens, rabbits, rats, hamsters and humans [17-19]. In this study, we investigated whether Thai RYR, Thai glutinous rice (*Oryza sativa* L. var. Niaw San-pah-tawng) fermented by *Monascus purpureus* CMU 002U, had the potential to lower the serum cholesterol and hepatic cholesterol levels of the diet-induced hypercholesterolemic rats. The mRNA expressions of key enzymes responsible for cholesterol biosynthesis and homeostasis, were determined, as was the lipid deposition of the liver. These effects offer a direct comparison between rats fed with a standard diet and rats fed with a high-fat cholesterol diet.

## Methods

### Red yeast rice preparation and extraction

Thai glutinous rice, fermented with *Monascus purpureus* CMU 002U (Red Yeast Rice), was formulated in the ‘Excellence in Sustainable Development of Biological Resource Laboratory’, Chiang Mai University, Chiang Mai, Thailand. A step by step preparation of the red yeast rice was executed, following a precise method developed by Chairote et al. [20]. At the end of cultivation, the product was dried at 65°C for 6 hours, and then ground in order to obtain an extract from the dried red yeast rice. One hundred grams of rice powder was soaked in 1,000 ml of 70% ethanol for a period of 24 hours. The extract was then filtered, to remove the residue, and evaporated using a vacuum rotary evaporator, in order to obtain the crude extract. The red yeast rice crude extract was then lyophilized and stored at -20°C, until further required. The residue was suspended in distilled water, and previously verified doses were accumulated for future experimentation.

### Animals

Male and female Wistar rats (*Rattus norvegicus*) were purchased from the ‘National Laboratory Animals Center’; a department of Mahidol University based at its Salaya campus, in the province of Nakhon Pathom, Thailand. The rats were housed in stainless steel cages, in a temperature controlled room having 12 hourly light and dark cycles, at temperatures between 24-26°C and a relative humidity of 55-60%. The rats were offered food and water *ad libitum*. These animal studies were approved by the Animal Care and Ethics Committee of the Biology Department, Faculty of Science, Chiang Mai University, Thailand. Food intake was recorded daily, with body weight also being recorded weekly.

### Acute toxicity study

To evaluate the safety of the red yeast rice extract used in this study, an acute toxicity test was initially conducted on female rats weighing between 100 and 120 g. This study was carried out as per the set guidelines of the Organization for Economic Co-operation and Development (OECD) [21]. The RYR extract used in this study was safe, up to a dosage level of 5000 mg/kg, *p.o*. Neither adverse effects on body weight and behaviors nor mortality was detected in any rats throughout the 14 day observation period.

### Diet and hypercholesterolemia induction

Four-week-old male rats weighing between 80 and 100 g were used in this study. Wistar rats were chosen as the experimental models since researches on hyperlipidemia and hypercholesterolemia have been studied extensively in this species. To induce hypercholesterolemia, after acclimatization comprising 7 days of a standard pellet diet (SP-diet), Thirty-two rats were switched to a modified high-fat content cholesterol diet (HFC-diet), as previously described by Kitamori et al. [22] for 45 days. The HFC-diet contained 68% standard diet (containing 3.06% fat), 12.5% palm oil, 12.5% lard, 5% cholesterol and 2% cholic acid. The nutritional components of the SP-diet and HFC-diet are summarized in Table 1.

**Table 1:** **Nutrition components of SP-diet and HFC-diet (weight %)** [23]

**Ingredients**	**SP-diet**	**HFC-diet**
**SP diet**	**100.0**	**68.0**
- Carbohydrate	46.5	31.62
- Crude protein	24.0	16.32
- Crude lipid	4.5	3.06
- Crude fiber	5.0	3.4
- Crude ash	10.0	6.8
- Moisture	10.0	6.8
- Palm oil	-	12.5
- Lard	-	12.5
- Cholesterol	-	5.0
- Cholic acid	-	2.0
**Total energy (kcal/100 g)**	**318.0**	**416.2**

The nutrition components are expressed as g/100 g of dry weight diet. SP-diet: standard pellet diet, HFC-diet: high fat and cholesterol diet.

### Experimental design

The rats were randomly assigned to six groups of 8 rats each and orally treated with RYR extract and rosuvastatin for 30 days according to the following regimes: Group I) SP-control: SP-dieted rats treated with distilled water at a volume of 2.0 ml/day. Group II) SP-2 g: SP-dieted rats treated with an RYR extract at a dose of 2.0 g/kg/day. Group III) HFC-control: HFC-dieted rats treated with distilled water at a volume of 2.0 ml/day. Group IV) HFC-1 g: HFC-dieted rats treated with RYR extract at a dose of 1.0 g/kg/day. Group V) HFC-2 g: HFC-dieted rats treated with RYR extract at a dose of 2.0 g/kg/day, and Group VI) HFC-rosuvastatin: HFC-dieted rats treated with rosuvastatin at a dose of 5.0 mg/kg/day. Rats in groups I-II were counted as “normocholesterolemic rats”, while those in groups III-VI were counted as “hypercholesterolemic rats”. The doses of RYR extract used in this study were based upon doses reported in previous studies [23,24]. At the end of the experimental period, all rats were sacrificed with diethyl ether. Blood samples were collected by cardiac puncture technique and prepared for serum biochemical assay. Vital organs (liver, heart, brain and kidneys) were immediately removed, cleaned and weighed. Portions of liver were fixed in Bouin’s solution for histopathological examination. For the liver mRNA determination, the liver portion was kept in TRIZOL reagent (Sigma-Aldrich Co. LLC, USA) then stored at -20°C, until needed. The rest of liver portion was freshly kept at -20°C for determination of liver lipid accumulation.

### Serum biochemical assay

The sera were separated from the blood by centrifugation at 3500 rpm for 10 minutes. The total cholesterol (TC), high density lipoprotein cholesterol (HDL-C) and low density lipoprotein cholesterol (LDL-C) were determined by using automated photometric systems, and with the cooperation of the ‘Medical Technology Clinic’, Faculty of Associated Medical Sciences, Chiang Mai University, Thailand.

Both the atherosclerotic index (AI) and the LDL-C/HDL-C ratio were calculated, using the following formulae: AI = (TC-HDL-C)/HDL-C and LDL-C/HDL-C ratio = LDL-C/HDL-C [25].

### Hepatic mRNA expressions and Real-time PCR analysis

Total RNA was extracted from the stored frozen liver tissues using an innuPREP RNA Mini Kit (Analytik Jena, Life Science, Jena, Germany) according to the manufacturer’s instructions. The cDNA was synthesized using protocol described by a Thermo Scientific RevertAid Reverse Transcriptase kit (Thermo Fisher Scientific, Waltham, MA, USA). 1 μl of diluted cDNA (1:5) was used in each real time-PCR, using a THUNDERBIRD SYBR qPCR Mix (Toyobo, Life Science, Osaka, Japan), and an Illumina Eco Real-Time PCR Instrument (Illumina, San Diego, CA, USA). The cycle condition was: 5 minutes at 95°C, followed by 45 cycles of incubation at 95°C for 30 minutes, 61°C for 30 seconds and, finally, 72°C for 20 seconds. The sequences of the primers used in this study were designed for rat 3-hydroxy-3-methyl-glutaryl-CoA reductase (HMG-CoA reductase) gene [GenBank No.: X55286], low-density lipoprotein receptor (LDL receptor) gene [GenBank No.: X13722], cholesterol 7 alpha-hydroxylase (CYP7A1) gene [GenBank No.: NM_012942], and glyceraldehyde-3-phosphate dehydogenase (GAPDH) [GenBank No.: NM_017008], as follows: HMG-CoA reductase forward 5-GGTGGTGGGACCAACCTTCT-3, reverse 5-CACGCCCCTTG AAC ACCTA-3: LDL receptor forward 5-CAGCCGATGCATTCCTGACT-3, reverse 5-AGTTCATCCGAGCCATTTTCA-3:CYP7A1 forward 5-CAAGTCAAGTGTCCCCCTCT AGA-3, reverse 5-ACTCAATATCATGTAGTGGTGGCAAA-3, and GAPDH forward 5-TGCCAAGTATG ATGACATCAAGAAG-3, reverse 5-AGCCCAGGATGCCCTTTAGT-3 [26]. The results were analyzed using software provided by the Eco Real-Time PCR System. Differences in mRNA expression were calculated using the 2^ΔΔct^ method, after normalizing to a GAPDH expression [27].

### Liver lipid extraction and determination

Lipids from livers were extracted using the Folch method [28]. One gram of tissue was ground in 20 ml of chloroform and methanol (2:1 v/v), and then sonicated for 30 minutes at room temperature. The aqueous layer was aspirated and discarded, and the chloroform layer was then evaporated until the residue was completely dry. The dried lipid layer was then dissolved with isopropyl alcohol containing 10% Triton-X100, and used to determine the TC concentration using commercial enzymatic kits, which had the same serum biochemical assay.

### Histopathological analysis

Histopathological analysis was conducted following the method of Buncharoen et al. [29]. The fixed liver tissues were dehydrated by progressively increased concentrations of ethanol, then passed through a xylene solution to clear the ethanol, and finally embedded in paraffin. Paraffin sections were then sliced into 6 μm thick by a rotary microtome. The tissue sections were stained with hematoxylin and eosin (H&E), and examined under a light microscope.

### Statistical analysis

The results were expressed by mean ± standard deviation (SD). Group means were compared using one-way analysis of variance (ANOVA), and the significance level was calculated using the Tukey HSD test. Values of *p* < 0.05 were considered to indicate statistical significance. All data were analyzed using SPSS 21.0 for Windows (SPSS Inc., Chicago, IL, USA).

## Results

### Acute toxicity study

Rats which have received an extract of red yeast rice at the dose of 5,000 mg/kg body weight did not exhibit any clinical signs of toxicity, changes in behavior, food or water consumption, or body weight, or death, immediately after oral administration and during the experimental period. There were no significant differences in body weight and food intake, and there were no histological alterations of the liver, heart, brain and kidneys in treated groups, when compared to the control group (data not shown). The extract could therefore, be considered to be safe.

### Effect of RYR administration on growth parameters and liver weight

As shown in Table 2, no significant difference in body weight gain was observed among all six groups. In addition, the daily food intake of hypercholesterolemic rats in HFC-control and HFC-1 g groups was significantly lower than those in the SP-groups (*p <* 0.05).

**Table 2:** **Effect of RYR extract on growth parameter and liver weight**

**Treatments groups**	**Body weight gain (g/30 days)**	**Food intake (g/rat/day)**	**Liver weight (g)**	**Relative liver weight (g/100 g BW)**
SP-control	77.50 ± 10.00^a^	20.69 ± 1.47^b^	10.70 ± 0.66^a^	2.53 ± 0.10^b^
SP-2 g	83.88 ± 7.85^a^	21.23 ± 2.57^b^	10.83 ± 0.73^a^	2.50 ± 0.21^b^
HFC-control	105.00 ± 17.56^a^	16.23 ± 2.00^a^	22.41 ± 3.79^c^	6.04 ± 0.57^a^
HFC-1 g	95.00 ± 17.32^a^	16.63 ± 2.46^a^	16.32 ± 2.51^b^	4.47 ± 0.33^ab^
HFC-2 g	103.75 ± 18.08^a^	18.72 ± 1.89^ab^	17.82 ± 2.37^b^	4.56 ± 0.42^ab^
HFC-rosuvastatin	101.43 ± 35.08^a^	17.83 ± 2.36^ab^	17.40 ± 2.25^b^	4.36 ± 0.39^ab^

Each value is the mean ± SD (n = 8). Values with different superscript letters (a,b,c) in the same column differ significantly (*p <* 0.05) by the Tukey HSD test.

The liver weights of hypercholesterolemic rats in all groups (HFC-groups) were significantly higher than those of normocholesterolemic rats (SP- groups) and HFC-control group showed the highest value of liver weigh. Administration of RYR extract at doses of 1.0 g/kg/day and 2.0 g/kg/day (HFC-1 g and HFC-2 g) significantly decreased liver wet weights by 25.79% and 20.48%, respectively, when compared to the HFC-control group, and the results were comparable to those of rats treated with rosuvastatin. Rats of the HFC-control group also displayed the highest relative liver weights. Although the relative liver weights of rats treated with RYR extract and rosuvastatin were not significantly different from those of the HFC-control group, a slight decrease in relative liver weights was observed. However, both liver wet weights and relative liver weights of normal rats treated with RYR extract (SP-2 g) were not significantly different from those of rats in the SP-control group.

### Effect of RYR administration on serum cholesterol

The serum cholesterol, including total cholesterol (TC), HDL-cholesterol, and LDL-cholesterol of rats in all groups are summarized in the Table 3.

**Table 3:** **Effect of RYR extract on serum cholesterol levels, atherosclerotic index, and LDL-C/HDL-C ratio**

**Treatment groups**	**Serum cholesterol (mg/dl)**			**Atherosclerotic index**	**LDL-C/HDL-C ratio**
	**Total cholesterol**	**HDL-C**	**LDL-C**		
SP-control	74.63 ± 11.31^a^	53.38 ± 7.78^a^	4.25 ± 3.11^a^	0.40 ± 0.06^a^	0.08 ± 0.01^a^
SP-2 g	74.00 ± 10.73^a^	52.13 ± 6.66^a^	3.50 ± 2.00^a^	0.42 ± 0.04^ab^	0.07 ± 0.04^ab^
HFC-control	150.29 ± 20.51^c^	48.00 ± 8.41^a^	58.67 ± 7.76^d^	2.13 ± 0.71^c^	1.22 ± 0.15^c^
HFC-1 g	102.86 ± 22.22^b^	54.43 ± 7.55^a^	36.71 ± 9.69^c^	0.89 ± 0.19^b^	0.67 ± 0.12^b^
HFC-2 g	95.63 ± 19.98^ab^	51.63 ± 7.95^a^	30.88 ± 12.10^bc^	0.85 ± 0.18^ab^	0.60 ± 0.18^ab^
HFC-rosuvastatin	85.57 ± 16.23^ab^	50.29 ± 9.91^a^	19.71 ± 5.91^b^	0.70 ± 0.10^ab^	0.39 ± 0.10^ab^

Each value is the mean ± SD (n = 8). Values with different superscript letters (a,b,c) in the same column differ significantly (*p <* 0.05) by the Tukey HSD test. HDL-C, high density lipoprotein cholesterol; LDL-C, low density lipoprotein cholesterol.

The concentration of serum TC of rats in the HFC-control group was significantly higher than those in the SP-control group, by 101.38% (*p <* 0.05). RYR extract at doses of 1.0 g/kg/day, 2.0 g/kg/day and rosuvastatin could significantly decrease TC concentrations by 31.56%, 36.37% and 43.06%, respectively, when compared to the HFC-control group (*p <* 0.05). Although the concentrations of serum TC of hypercholesterolemic rats treated with RYR extract at the dose of 2.0 g/kg/day and rosuvastatin were slightly higher than those of normocholesterolemic rats (SP-groups), the statistical differences between those three groups were not evidenced.

The HDL-C concentrations of rats in all groups remained at similar levels, while the LDL-C concentration in the HFC-control became significantly higher than that of the SP-control group (*p <* 0.05). LDL-C levels of hypercholesterolemic rats administered with RYR extract at doses of 1.0 g/kg/day and 2.0 g/kg/day were, however, significantly lower than those of HFC-control group by 37.42% and 47.37%, respectively (*p <* 0.05). The hypocholesterolemic effect of RYR extract was found to be comparable to that of rosuvastatin.

The normal rats treated with RYR 2.0 g/kg/day (SP-2 g) presented similar concentrations of all serum cholesterol, to those of normal control rats fed with a SP-diet (SP-control).

### Effect of RYR administration on atherosclerotic index and LDL-C/HDL-C ratio

The atherosclerotic index (AI) is an index used to predict the risk of atherosclerosis in hypercholesterolemic patients, and is expressed by the ratio (TC-HDL-C)/HDL-C. It was found that the AI of hypercholesterolemic rats in the HFC-1 g, HFC-2 g and HFC-rosuvastatin groups were all reduced by approximately 60.63%, 61.99% and 67.87%, respectively (*p <* 0.05), when compared to the HFC-control group. Similarly, rats treated with RYR extract at both doses used, and with rosuvastatin, displayed a significantly lower LDL-C/HDL-C ratio, a predictive indicator of cardiovascular diseases than the HFC-control group, by 45.96%, 52.41% and 68.54%, respectively (*p <* 0.05). The AI and LDL-C/HDL-C ratio of normocholesterolemic rats in both the SP-control and SP-2 g groups were extremely low, and no difference in both indices was observed between these 2 groups (Table 3).

### Effect of RYR administration on hepatic mRNA expressions

**Figure 1:**
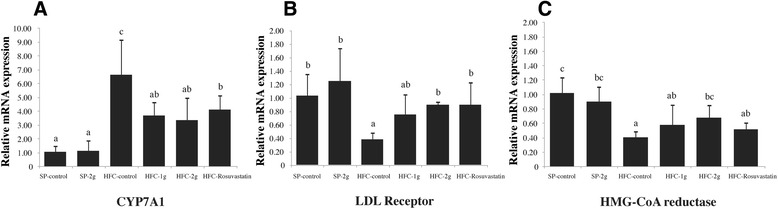
**Hepatic mRNA expressions of cholesterol metabolism**-**related genes.** Hepatic cholesterol 7 alpha-hydroxylase (CYP7A1) **(A)**, LDL receptor **(B)**, and HMG-CoA reductase **(C)** in male rats, fed with SP-diet and HFC-diet with red yeast rice extract and rosuvastatin for 30 days. Values were normalized to reference gene GAPDH, and are expressed relative to the control level (SP-control). Data are expressed as mean ± standard deviation (SD) (n = 8). Each value is the mean ± SD (n = 8). Values with different superscript letters (a,b,c) in the each column differ significantly (*p* < 0.05) by the Tukey HSD test.

The mRNA expressions of cholesterol homeostasis, including CYP7A1, the LDL receptor and HMG-CoA reductase in hypercholesterolemic rats are presented in Figure 1. The mRNA expression of CYP7A1 of hypercholesterolemic rats in all groups was higher than that of SP-control rats. Nevertheless, significant decrease of CYP7A1 expression (*p* < 0.05) was observed in hypercholesterolemic rats treated with RYR, (HFC-1 g and HFC-2 g) and rosuvastatin (HFC-rosuvastatin). The mRNA expression of the LDL receptors in the HFC-control group was, remarkably, the lowest, whereas in the HFC-2 g and HFC-rosuvastatin groups it was significantly higher than in the HFC-control group (*p* < 0.05). Likewise, the mRNA expression of HMG-CoA reductase in HFC-control group was also significantly lower than that of SP-control group (*p* < 0.05). Nevertheless, HFC-2 g could significantly increase the expression of HMG-CoA reductase when compared with HFC-control group (*p <* 0.05), while rosuvastatin did not show this result. No significant difference in all mRNA expressions was observed between the SP-control and SP-2 g groups.

### Effect of RYR administration on hepatic lipids

The lipid accumulations in the livers of the SP-diet and HFC-diet fed rats were investigated, by measuring the liver total lipid (TL) and total cholesterol (TC) levels, as shown in Table 4. The TL and TC levels of the HFC-diet fed rats were significantly higher than those of the normal rats fed with a SP-diet. RYR extract at doses of 1.0 g/kg/day and 2.0 g/kg/day and rosuvastatin could significantly decrease liver TL concentrations of hypercholesterolemic rats by 26.81%, 28.05% and 27.49%, respectively and decrease liver TC concentrations by 31.22%, 50.86% and 35.26%, respectively (*p* < 0.05), when compared to the HFC-control group. Moreover, TC concentrations of hypercholesterolemic rats treated with RYR extract and rosuvastatin were comparable to those of normocholesterolemic rats.

**Table 4:** **Effect of RYR extract on hepatic total lipid and total cholesterol levels**

**Treatment groups**	**Hepatic lipid (mg/g liver)**	
	**Total lipid**	**Total cholesterol**
SP-control	41.16 ± 6.75^a^	6.00 ± 0.95^a^
SP-2 g	38.40 ± 5.34^a^	5.44 ± 0.89^a^
HFC-control	187.09 ± 5.84^c^	9.20 ± 0.50^b^
HFC-1 g	136.93 ± 12.93^b^	6.33 ± 1.66^a^
HFC-2 g	134.60 ± 9.89^b^	4.52 ± 1.96^a^
HFC-rosuvastatin	135.66 ± 11.75^b^	5.96 ± 1.68^a^

Each value is the mean ± SD (n = 8). Values with different superscript letters (a,b,c) in the same column differ significantly (*p <* 0.05) by the Tukey HSD test.

### Effect of RYR administration on macroscopic and microscopic structures of the liver

**Figure 2:**
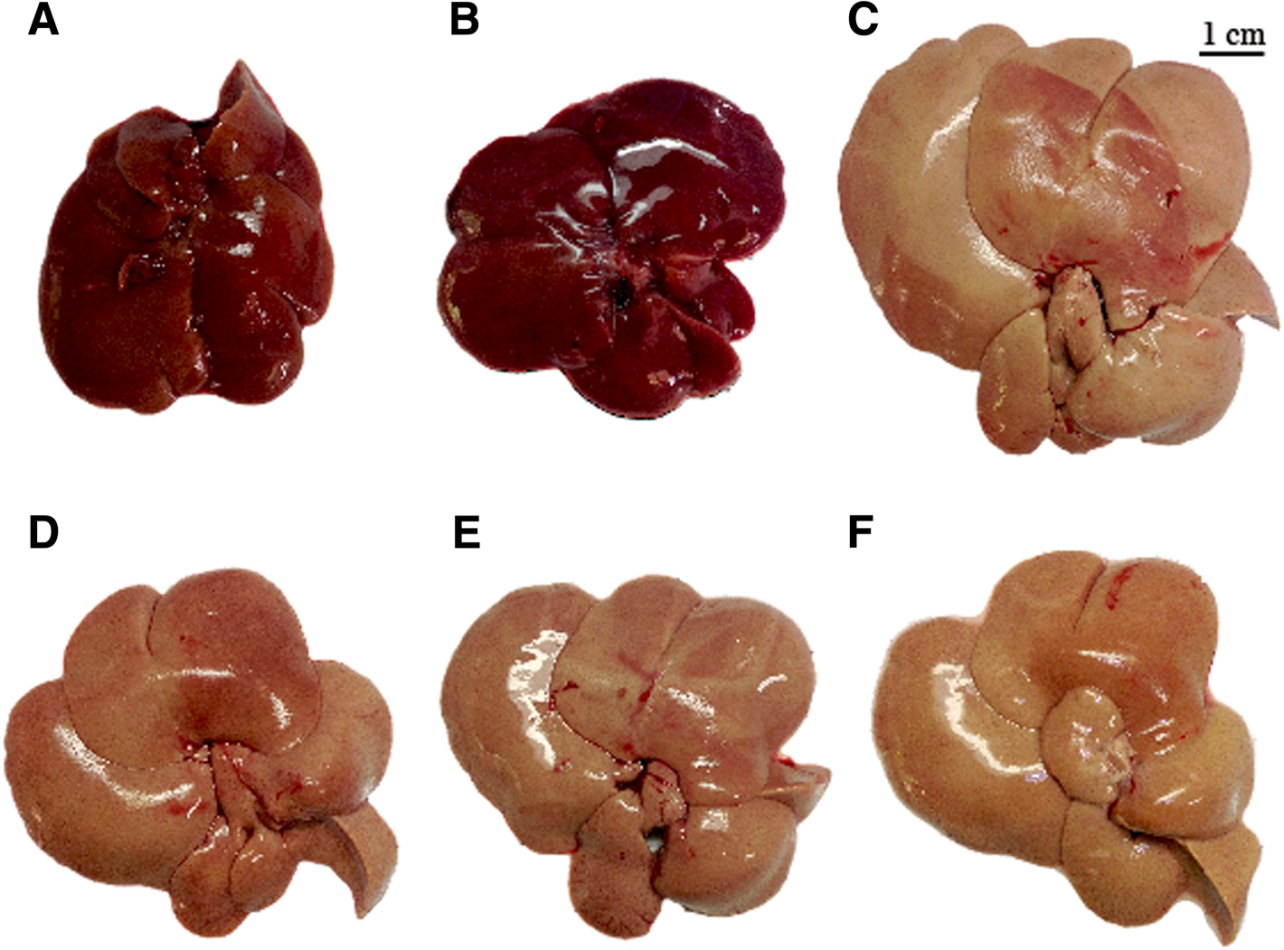
**Macroscopic photo of liver.** Livers of normal rats treated with distilled water **(A)** and 2.0 g/kg/day of RYR extract **(B)**, and livers of hypercholesterolemic rats treated with distilled water **(C)**, 1.0 g/kg/day of RYR extract **(D)**, 2.0 g/kg/day of RYR extract **(E)** and 5.0 mg/kg/day of rosuvastatin **(F)**, for 30 days.

Gross observation revealed that all hypercholesterolemic rats had developed fatty livers. The liver sizes of all rats fed with a high-fat and cholesterol diet were larger than those of normal rats fed with an SP-diet, and they also became yellow-brown. The liver sizes of hypercholesterolemic rats treated with RYR extract and rosuvastatin were equal to those of the normal control rats (SP-control). The liver sizes and colors of the normal rats treated with RYR extract (SP-2 g) were not different from those of the normal control rats (SP-control) (Figure 2).

**Figure 3:**
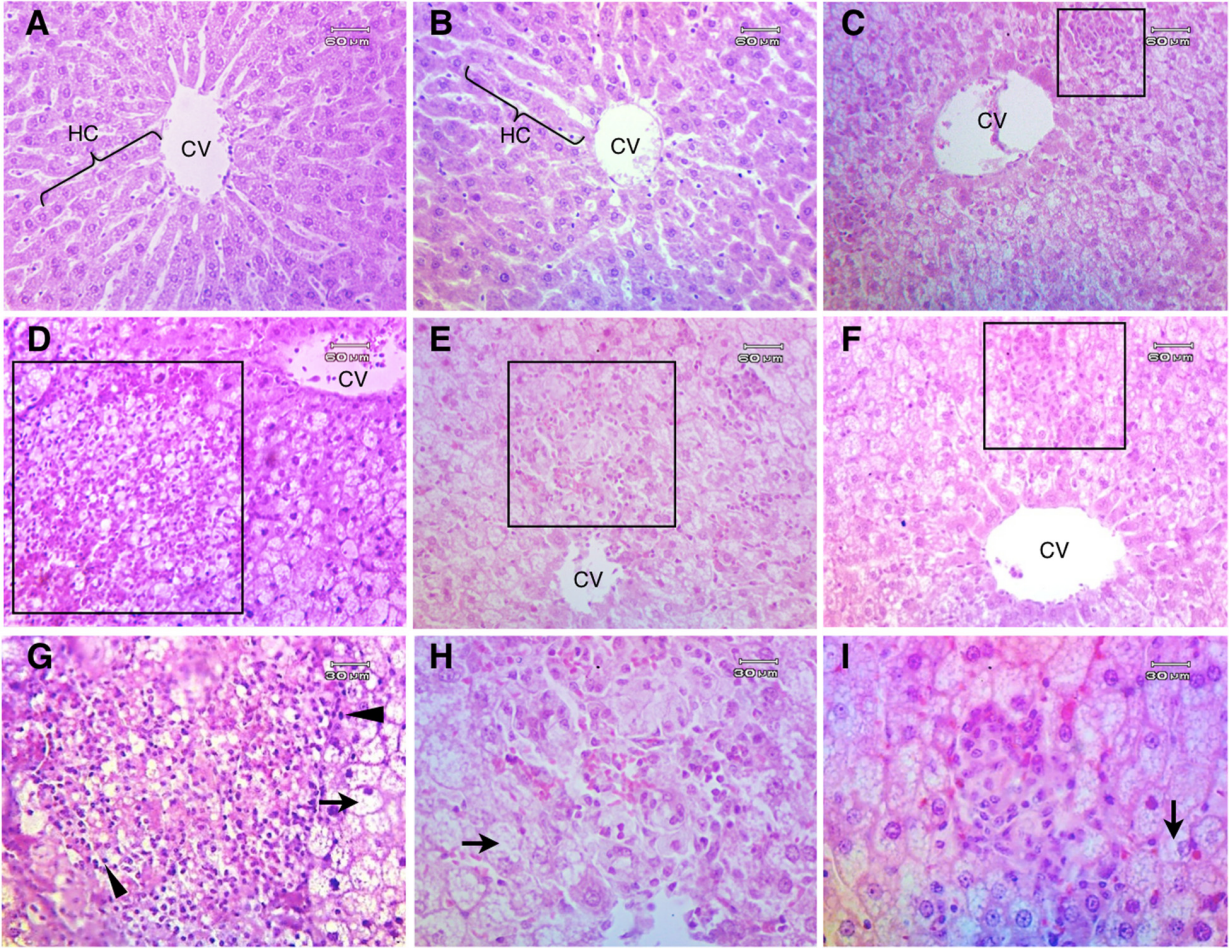
**Microscopic characters of liver tissue.** Liver tissue of normal rats treated with distilled water **(A)** and 2.0 g/kg/day of RYR extract **(B)**, and liver tissue from hypercholesterolemic rats treated with 5.0 mg/kg/day of rosuvastatin **(C)**, distilled water **(D, G)**, 1.0 g/kg/day of RYR extract **(E, H)**, and 2.0 g/kg/day of RYR extract **(F, I)**. Original magnifications were 100x **(A-F)** and 200x **(G-I)**. Lipid deposition in hepatocyte (steatosis) (arrow), steatosis with inflammatory cell infiltrations (steatosis hepatitis) (quadrilateral) and nuclear condensation (head arrow) were observed in the liver of hypercholesterolemic rats **(D-I)**. No remarkable damage was detected in SP and SP-2 g **(A, B)**. CV, central vein; HC, hepatic cord (**H** & **E**).

Microscopically, fat droplets were found deposited in the hepatocytes of all rats fed with an HFC-diet. In the HFC-control group, the presence of steatosis with inflammatory cell infiltrations and nuclear condensation was observed in the large area of acinar zone 3 of the liver, while the HFC-1 g group showed a moderate amount of steatosis, with inflammatory cell infiltrations in the small area of acinar zone 3. The livers of the HFC-2 g and HFC-rosuvastatin groups displayed steatosis with inflammatory cell infiltrations to a much lesser extent than the HFC-control and HFC-1 g groups, with most of the nucleus remaining centrally located. Furthermore, in the livers of rats fed on a SP-diet (SP-control and SP-2 g) the morphology of hepatocytes still displayed normal architecture around the central veins, still arranged in cords. No sign of a liver displaying both steatosis and hepatitis was observed (Figure 3).

## Discussion

RYR is not only valued for cuisine in China and East Asia, it is also used in traditional medicine to improve digestion, spleen function, blood circulation and to resolve blood stasis. RYR contains many compounds which are believed to help lower blood lipid levels. Those compounds include: monacolin (mainly HMG-CoA reductase inhibitors, or monacolin K), palmitic acid, linoleic acid, oleic acid and stearic acid [11,16]. Although the hypolipidemic effects of RYR extract have been well documented, there have been insufficient reports about RYR from local Thai rice. In this study, we demonstrate the potential of Thai RYR to reduce serum cholesterol levels, when produced by fermentation of Thai glutinous rice (*Oryza sativa* L. var. Niaw San-pah-tawng) by *Monascus purpureus* CMU 002U.

Hypercholesterolemia could be in both inherited and non-inherited forms. Familial hypercholesterolemia, a genetic disorder caused by the mutations of genes that encode LDL receptor, occurs less common than the non-inherited form [2,7]. Due to the worldwide increase in incidence of diet-induced hypercholesterolemia, we decided to test the efficacy of Thai RYR extract in diet-induced hypercholesterolemia Wistar rats. The similarities between Wistar rat and human in the aspects of physiology and metabolism make this species the promising animal models for several metabolic syndromes, including hyperlipidemia and hypercholesterolemia [10,30,31]. Using high-fat cholesterol diets to induce hypercholesterolemia in rats is a popular and successful method much used in research, and was also used in this study. It led to enhanced serum TC and LDL-C levels in the HFC-control group, measured at 150.29 mg/dl and 74.63 mg/dl, respectively. This indicates that the HFC-diet had successfully induced hypercholesterolemia in the rats in this study. In reference to our results, the major change observed in our experiment was the reduction of serum TC and LDL-C levels in rats fed with RYR 1.0 and 2.0 g/kg/day. The serum TC decreased by 31.55% and 36.36%, LDL-C decreased by 37.42% and 47.36% in the HFC-1 g and HFC-2 g groups, respectively, compared with the HFC-control group (150.29 mg/dl in TC and 58.67 mg/dl in LDL-C). These were confirmed by the positive control group (HFC-rosuvastatin), in which the serum TC and LDL-C were decreased from the HFC-control group by 43.06% and 66.40%, respectively.

According to a previous study, in which RYR was administered to rabbits for 200 days, the serum TC was 25% and 40% lower in the rabbits fed with 0.4 g/kg/day or 1.35 g/kg/day of red yeast rice, respectively, and there was also lower levels of serum LDL-C and triglyceride [12]. Additionally, upon examination of Chinese RYR consumption (2.4 g/day) in an American population with hypercholesterolemia, the serum TC concentration decreased from 254.0 mg/dl to 208.0 mg/dl in 8 weeks, and LDL-C and TG levels also decreased [23]. When Indian RYR was evaluated in rat models by administering RYR at doses of 1.2 mg/kg and 2.4 mg/kg/day to the rats for 30 days, the extract at both doses could maintain the lipid profile (TC, TG, HDL, LDL-C and VLDL-C) of the rats at near normal status [30]. Also, the administration of RYR extract at doses of 0.4 g/kg/day and 0.8 g/kg/day to the rabbits for 30 days could significantly lower their serum total cholesterol (TC) concentrations [17]. It is unclear that which substance(s) take the major role in lowering cholesterol level, either only monacolin K or a combination of monacolin K with other substances [11]. However, many researchers have succeeded in using RYR to lower lipid levels in animal models and humans, due to the presence of the same cholesterol synthesis inhibitor, monacolin K [17,32]. A liver cholesterol biosynthetic pathway started with HMG-CoA reductase, an enzyme that catalyzes the conversion of HMG-CoA to mevalonate [33], which is the first key chemical in the biosynthetic pathway leading to cholesterol as the final product. Mevalonate activity is reduced by the inhibitor known as the HMG-CoA reductase inhibitor or monacolin K, a substance found in RYR. This is the specific inhibitor in the reaction of HMG-CoA reductase because monacolin K has the same chemical structure as the ‘statin drugs’, a group of drugs used in hypercholesterolemia treatment and potent inhibitors of 3-hydroxy-3-methylglutaryl-coenzyme A (HMG-CoA) reductase [34].

Indeed, the target of hypercholesterolemic therapies is a reduction of the atherosclerotic index value and the LDL-C/HDL-C ratios, along with a decrease of the major lipids, which are key risk factors for cardiovascular disease, particularly total cholesterol and LDL-C [26]. This is consistent with the obtained data. The HFC-control group in this experiment had a higher risk of atherosclerosis and cardiovascular diseases than any other group. Furthermore, in the rats treated with RYR the index value of both doses decreased, such that the results of the primary prevention trials with RYR have demonstrated that lowering serum cholesterol and LDL-C cholesterol can significantly reduce the risk of cardiovascular events [35,36].

Generally, the liver is considered to be the primary organ responsible for cholesterol homeostasis maintenance, by regulating cholesterol synthesis and uptake as well as cholesterol excretion into bile acids [37]. Gene regulation mechanism implicated in cholesterol homeostasis has been well-documented and the three genes, HMG-CoA reductase, LDL receptor and CYP71, were proposed as the key genes for the synthesis, absorption and degradation of hepatic cholesterol, respectively. The decrease of mRNA expression of HMG-CoA reductase and LDL receptor and the increase of that of CYP71 were found in animals with high level of plasma cholesterol condition [10,38,39]. The results of mRNA expression of HMG-CoA reductase, LDL receptor and CYP71 in diet-induced hypercholesterolemic rats (HFC-control group) which correlated with the reports of the previous study could support the importance of those 3 genes in cholesterol homeostasis. Attempts have been made to explore the molecular mechanism(s) underlying the effect of cholesterol lowering agents and different results have been obtained from different researches [26,40]. Those different results indicated the different mechanisms of their hypocholesterolemic effect. In this study, the ability of RYR extract at 2.0 g/kg/day to enhance mRNA expression of HMG-CoA reductase and LDL receptor reflect its potential to adjust the rate of cholesterol synthesis and absorption. HMG-CoA reductase is the regulatory enzyme of hepatic cholesterol biosynthesis. The enzyme levels are suppressed by exogenous cholesterol and degradation of low density lipoproteins (LDL-C) via the up-regulation of the LDL receptor [31]. CYP7A1 is a gene involved in the biosynthetic pathway of bile acids from cholesterol in liver for excretion into bile [41]. Kawakami et al. [42] reported that the increased expression of CYP7A1 is the mechanism of hypocholesterolemic action that promoted excretion of cholesterol and bile acids. In the present study, the mRNA expressions of CYP7A1 of diet-induced hypercholesterolemic rats treated with RYR extract and rosuvastatin were lower than those of HFC-control group. This result is not consistent with previous studies, which showed that the expression of CYP7A1 were up-regulated in rats treated with puerarin [40]. The down regulation of CYP7A1 expression by RYR extract implied that cholesterol excretion was not the mechanism for its hypocholesterolemic effect. Therefore, the decreasing of blood cholesterol concentration by RYR extract was likely to be due to its ability to promote the biosynthesis and absorption of blood cholesterol by increasing the hepatic HMG-CoA reductase and LDL receptor expression.

In addition, this study clearly showed that the liver weights of all HFC-diet fed rats (HFC-control, HFC-1 g, HFC-2 g, HFC-rosuvastatin) were significantly higher than those of SP-diet fed rats (SP-control and SP-2 g). Our data exhibited that in rats fed only an HFC-diet and treated with RYR extract, at 1.0 g/kg/day and 2.0 g/kg/day, their liver weight decreased by 25.79% and 20.48%, respectively. The liver weight results were consistent with the concentrations of lipid in the liver. The HFC-control group had the highest TC concentrations compared to all groups, significantly higher than those of rats treated with RYR 1.0 g/kg/day and 2.0 g/kg/day. The results indicated that high lipid and cholesterol foods led to the development of hepatic steatosis conditions, or fatty liver [43], where the hepatocyte is in retention of fat within the cell. The imbalance between acquisition by uptake of non-esterified fatty acids from the plasma and by *de novo* lipogenesis and triglyceride disposal by fatty acid oxidation and by the secretion of triglyceride-rich lipoproteins was suggested as the mechanism underlying lipid accumulation in hepatocytes [44]. The result of hepatic lipid accumulation can be confirmed by microscopic images of the liver in Figure 3. These images display the large vacuoles of fat accumulated within hepatocytes (steatosis), and inflammatory cell infiltrations that were observed in the HFC-diet fed rats. The large area of steatosis with inflammatory cell infiltrations was observed in all HFC-diet fed rats, especially in the HFC-control group (Figure 3D,G). Nevertheless, HFC-diet fed rats treated with RYR at 1.0 and 2.0 g/kg/day exhibited a smaller area of steatosis with inflammatory cells than the HFC-control group. Interestingly, the capacity of the extract at the dose of 2.0 g/kg/day to reduce liver steatosis was nearly comparable to rosuvastatin, a synthetic hypocholesterolemic drug. When this steatosis becomes associated with inflammation, it is called steatosis hepatitis, and forms chronic liver disease [45]. This feature can be classified histologically, by lesions that differ from the steatosis and inflammation, into 3 types: type 1 (mild) the amount of steatosis and inflammation is less than 33% in the acinar zone 3 area: type 2 (moderate) is observed at 33%-66%: and more than 66% is type 3 (severe) steatosis [46,47]. According to this classification, the livers of the HFC-control group were already classified as types 3, while the hypercholesterolemic rats treated with RYR 1.0 g/kg/day and 2.0 g/kg/day were already classified as types 2 and 1, respectively. From our findings, the extract of Thai RYR can help reduce the development of severe steatosis and chronic liver disease.

## Conclusion

The experimental data suggest that Thai RYR, Thai glutinous rice fermented by *Monascus purpureus* CMU 002U, could potentially decrease serum total cholesterol and LDL-C levels, and also reduce atherosclerosis and cardiovascular diseases. Furthermore, this RYR could decrease lipid accumulation in the liver, thereby delaying the onset of hepatic steatosis. The success of this study could lead to future research for developing cholesterol lowering products from a local rice of Thailand.

## Abbreviations

RYR: Red yeast rice
TC: Total cholesterol
TG: Triglyceride
LDL-C: Low density lipoprotein cholesterol
HDL-C: High density lipoprotein cholesterol
HMG-CoA reductase: 3-hydroxy-3-methyl-glutaryl-CoA reductase
HMGR: 3-hydroxy-3-methyl-glutaryl-CoA reductase gene
LDLR: Low-density lipoprotein receptor gene
CYP7A1: Cholesterol 7 alpha-hydroxylase gene
GAPDH: Glyceraldehyde-3-phosphate dehydrogenase
SP-diet: Standard pellet diet
HFC-diet: High-fat and cholesterol diet
